# A Transcriptomic Study Reveals That Fish Vibriosis Due to the Zoonotic Pathogen *Vibrio vulnificus* Is an Acute Inflammatory Disease in Which Erythrocytes May Play an Important Role

**DOI:** 10.3389/fmicb.2022.852677

**Published:** 2022-04-01

**Authors:** Carla Hernández-Cabanyero, Eva Sanjuán, Felipe E. Reyes-López, Eva Vallejos-Vidal, Lluis Tort, Carmen Amaro

**Affiliations:** ^1^Instituto Universitario de Biotecnología y Biomedicina (BIOTECMED), Universitat de València, Valencia, Spain; ^2^Centro de Biotecnología Acuícola, Facultad de Química y Biología, Universidad de Santiago de Chile, Santiago, Chile; ^3^Department of Cell Biology, Physiology, and Immunology, Universitat Autònoma de Barcelona, Bellaterra, Spain; ^4^Facultad de Medicina Veterinaria y Agronomía, Universidad de Las Américas, Santiago, Chile

**Keywords:** *Vibrio vulnificus*, zoonotic pathogen, blood, erythrocytes, European eel, host-pathogen relationship, immune response

## Abstract

*Vibrio vulnificus* is a marine zoonotic pathogen associated with fish farms that is considered a biomarker of climate change. Zoonotic strains trigger a rapid death of their susceptible hosts (fish or humans) by septicemia that has been linked to a cytokine storm in mice. Therefore, we hypothesize that *V. vulnificus* also causes fish death by triggering a cytokine storm in which red blood cells (RBCs), as nucleated cells in fish, could play an active role. To do it, we used the eel immersion infection model and then analyzed the transcriptome in RBCs, white BCs, and whole blood using an eel-specific microarray platform. Our results demonstrate that *V. vulnificus* triggers an acute but atypical inflammatory response that occurs in two main phases. The early phase (3 h post-infection [hpi]) is characterized by the upregulation of several genes for proinflammatory cytokines related to the mucosal immune response (*il17a/f1* and *il20*) along with genes for antiviral cytokines (*il12β*) and antiviral factors (*ifna* and *ifnc*). In contrast, the late phase (12 hpi) is based on the upregulation of genes for typical inflammatory cytokines (*il1β*), endothelial destruction (*mmp9 and hyal2*), and, interestingly, genes related to an RNA-based immune response (*sidt1*). Functional assays revealed significant proteolytic and hemolytic activity in serum at 12 hpi that would explain the hemorrhages characteristic of this septicemia in fish. As expected, we found evidence that RBCs are transcriptionally active and contribute to this atypical immune response, especially in the short term. Based on a selected set of marker genes, we propose here an *in vivo* RT-qPCR assay that allows detection of early sepsis caused by *V. vulnificus.* Finally, we develop a model of sepsis that could serve as a basis for understanding sepsis caused by *V. vulnificus* not only in fish but also in humans.

## Introduction

Fish vibriosis encompasses a group of diseases with common clinical signs caused by different genus Vibrio species (Amaro et al., 2020). Among these species, *Vibrio vulnificus* stands out as the only one linked to zoonotic cases acquired through contact with diseased fish, mainly farmed fish (Amaro et al., 2015; Oliver, 2015). Moreover, it is the only one that can cause rapid death by septicemia in both humans and fish (Ceccarelli et al., 2019; Amaro et al., 2020).

The severity of outbreaks caused by *V. vulnificus* in fish farms is highly dependent on water temperature since the highest mortality rates occur at temperatures above 25°C (Amaro et al., 1995). This temperature dependence explains why this vibriosis (hereafter Vv-vibriosis) mainly affects fish reared above 22°C, as well as why clinical cases in humans and animals are increasing with global warming (Ceccarelli et al., 2019; Amaro et al., 2020). Part of the reason for this dependence is that an increase in temperature above 22°C significantly increases the transcription of several pathogen genes related to colonization and resistance to innate immunity in fish, thus favoring disease transmission and unbalancing the host-pathogen relationship toward the pathogen (Hernández-Cabanyero et al., 2020). These data correlate with field data and underline the relevance of *V. vulnificus* as a biological barometer of climate change (Baker-Austin et al., 2012, 2018).

Vv-vibriosis differs from other vibriosis in that death from septicemia occurs very quickly and without the pathogen reaching as high numbers in the blood or tissues as it does in other vibriosis (Valiente and Amaro, 2006; Valiente et al., 2008a). Previous studies in eels infected by immersion showed that the pathogen infects animals through water, colonize the gill and intestinal epithelium and cause local inflammation that favors its entry into the blood (Marco-Noales et al., 2001; Callol et al., 2015a). Furthermore, a series of additional studies demonstrated that once in the bloodstream, the pathogen produces a series of iron-regulated proteins that allow it to resist innate immunity and survive (Pajuelo et al., 2016; Hernández-Cabanyero et al., 2019). However, although toxins and exoenzymes that could cause cell death and/or tissue injury are known (Jeong and Satchell, 2012; Lee et al., 2013), is less clear the mechanism by which such a small number of bacteria triggers rapid death by sepsis. Murciano et al. (2017) shed light on how this bacterium could cause rapid death by sepsis. The authors infected mice by intraperitoneal injection with a zoonotic strain. They showed that the animal’s death was related to an early cytokine storm triggered by the pathogen. However, although the mouse is the animal model used to study human vibriosis, it is neither a natural host for *V. vulnificus* nor is injection a natural route of infection. Therefore, it would have to be shown that the pathogen triggers a cytokine storm by using one of its natural animal hosts infected by the natural route.

Given the above, the main objective of this study was to demonstrate that *V. vulnificus* causes an early cytokine storm in fish. To do so, we infected eels by immersion (Amaro et al., 1995) with the same strain used by Murciano et al. (2017). We analyzed the transcriptome in blood using an eel-specific microarray platform containing probes for thousands of immune-related genes (Callol et al., 2015b). Since fish red blood cells (RBCs) are nucleated cells involved in defense against viruses (Workenhe et al., 2008; Morera et al., 2011; Dahle et al., 2015; Nombela et al., 2017; Nombela and Ortega-Villaizan, 2018), we also considered analyzing the RBCs-associated transcriptome. Accordingly, we studied and compared the transcriptome associated with RBCs, white BCs (WBCs), and whole blood (B) at 0-, 3- and 12-h post-infection (hpi). We then validated the results obtained by RT-qPCR and performed a series of functional confirmatory assays. Our results suggest that *V. vulnificus* triggers an acute but atypical inflammatory response in two main phases. The early phase (detectable at 3 hpi) is characterized by the upregulation of important genes for proinflammatory cytokines related to the mucosal immune response (*il17a/f1* [in RBCs] and *il20* [in RBCs and WBCs]) along with antiviral cytokine genes (*il12β* [in both cell types]) and antiviral factors (*ifna* and *ifnc* [in WBCs only]). The late phase (detectable at 12 hpi) is characterized by the upregulation of genes for typical inflammatory cytokines (*il1β*), endothelial destruction (*mmp9* and *hyal2*), and genes related to an RNA-based immune response (*sidt1*), all of them detected in B samples. Functional assays revealed significant proteolytic and hemolytic activity in serum at 12 hpi that would explain the hemorrhages characteristic of this septicemia. As expected, we found evidence that RBCs are transcriptionally active and may contribute to this atypical immune response, especially in the short term. We also selected a series of marker genes and validated *in vivo* an RT-qPCR assay for early detection of Vv-vibriosis. Finally, we developed a model of septicemia that could serve as a basis for understanding sepsis caused by *V. vulnificus* not only in fish but also in humans.

## Materials and Methods

### Animal Maintenance

Adult European eels (*Anguilla anguilla*) of around 20 g (50% lethal dose [LD_50_] determination) or 100 g (sample collection for transcriptomic experiments) of body weight were purchased from a local eel farm (Valenciana de Acuicultura SA, Spain) that does not vaccinate against *V. vulnificus*. Eel maintenance and all the experiments described above were performed at 28°C in 180-liter tanks containing either 60 (infection experiments) or 120 L (the rest of experiments and animal maintenance) of saline water (SW, 1.5% NaCl, pH 7) with a system of aeration and filtration in the facilities of the Central Service for Experimental Research (SCSIE) of the University of Valencia (Spain).

### Bacterial Strain, Growth Media, and Conditions

The *V. vulnificus* strain CECT 4999 (Spanish Type Culture Collection; hereafter R99), which was isolated from a diseased eel in Spain (Lee et al., 2008), was used in this study. It was routinely grown in Tryptone Soy Agar or Luria-Bertani broth, both supplemented with 1% NaCl (TSA-1 and LB-1, respectively) with gentle agitation (100 rpm), at 28°C for 18 h to reach a concentration of 10^9^ colony forming units (CFU)/ml for bath infection. Bacterial concentration was checked before and after bath infection by drop-plate counting in TSA-1 plates (Hoben and Somasegaran, 1982). The bacterial strain was stored in LB-1 plus glycerol (20%) at −80°C.

### *In vivo* Bacterial Challenge, Sample Collection, and Preparation

Before the transcriptomic experiments, the LD_50_ of R99 to the eel stock was determined by immersion challenge, according to Amaro et al. (1995). Briefly, groups of 6 eels of 20 g were immersed in tanks containing either seawater (SW; control group) or serial decimal concentrations of R99 strain in SW (from 1 × 10^8^ to 1 × 10^5^ CFU/ml) for 1 h. Eels were then transferred to new tanks containing fresh SW and monitored for 1 week. Moribund animals were microbiologically analyzed to confirm that they were infected with *V. vulnificus* (liver sampling in TSA-1 and TCBS, followed by serological confirmation), and LD_50_ was calculated according to Reed and Muench (1938). For transcriptomic experiments, eels of 100 g were distributed into two groups, the tested (*n* = 24 individuals) and the control group (*n* = 6 individuals). Individuals were then immersed either in an infective bath containing 2 × 10^6^ CFU of the strain R99 (the previously estimated LD_50_; tested group) or in sterilized SW (control group). After 1 h of immersion, fish were transferred separately into new tanks and kept under constant conditions until sampling. We selected as sampling points, time zero (0 hpi; used as another control for the analysis), 3 hpi [as the early time at which most *V. vulnificus* virulence factors are expressed *in vivo* (Lee et al., 2013; Callol et al., 2015b; Murciano et al., 2017; Hernández-Cabanyero et al., 2019)], and 12 hpi [the average time at which eels start to die (Amaro et al., 2015)]. Six live eels were randomly sampled at the selected times. Prior sampling, eels were anesthetized with MS222 (50 mg/l), and around 2.5 ml of blood per individual was extracted from the caudal vein with heparinized syringes. Bled eels were then sacrificed using an overdose of MS222 (150 mg/l). Next, a volume of 0.5 ml of the sampled blood was used for bacterial drop-plate counting on TSA-1 and blood cell counts (RBCs and WBCs), a volume of 1 ml was used as a whole blood sample (B), and the rest was processed to get RBCs and WBCs samples. To this end, blood was centrifuged at 800 × *g* for 5 min. Serum was removed from cells and stored at −80°C until use (for fuctional assays, see below). The pelleted cells were washed with 1 ml of Phosphate Buffered Saline (PBS, pH 7), centrifuged again at 800 × *g* for 5 min, and the final pellet was resuspended in the same volume of PBS. Then, a density gradient separation was carried out by mixing the suspension with Ficoll^®^-Paque Premium (Sigma-Aldrich; vol:vol) and centrifugation at 720 × *g* for 30 min. RBCs and WBCs layers were collected and washed in PBS. We assured that the samples were not contaminated with other cellular populations by observation under the microscope. Finally, the different samples were treated with 1 ml of NucleoZOL (Macherey-Nagel) and stored at −80°C until use. All the *in vivo* experiments were performed in triplicate.

### RNA Extraction, Microarray Hybridization, and Data Analysis

Total RNA (from eels B, RBCs, and WBCs obtained at 0, 3, and 12 hpi) was extracted with NucleoZOL (Macherey-Nagel) following the manufacturer’s instructions. Possible contaminating DNA was eliminated using TURBO^™^ DNase (Ambion) and then, RNA was cleaned with RNA Cleanup and Concentration Micro Kit RNA (Thermo Scientific) according to the manufacturer’s instructions. RNA integrity and quality were verified with a 2,100 Bioanalyzer (Agilent), and only high-quality samples (RNA Integrity Number [RIN] ≥ 7.5) were selected and used for hybridization with the microarray.

For hybridization, it was used a custom eel-specific microarray platform that contains 42,403 probes (3 per target) of 60-oligonucleotide in length (accession number GPL16775) corresponding to each one of the ORFs identified in the eel immune-transcriptome determined by Callol et al. (2015b). Since the eel genome was not available at the moment the microarray was designed, the eel immune-transcriptome was annotated by similarity with other genomes, searching sequence homologies against NCBI’s non-redundant protein, and NCBI’s redundant nucleotide database by bestBLAST iterative methodology (Callol et al., 2015b). Therefore, the microarray genes in this work refer to those annotated genes. General procedures to obtain labeled cDNA were performed as previously described by Callol et al. (2015b).

Microarray data were extracted from raw images with Feature Extraction software (Agilent technologies). Quality reports were generated and checked for each array. Extracted raw data were imported and analyzed with Genespring GX 14.5 software (Agilent technologies). The 75% percentile normalization was used to standardize arrays for comparisons. All samples were analyzed at gene level using a relative analysis, comparing each sample against a reference sample (0 hpi sample of each cell type). Supplementary Figure S1 summarizes the experimental design and all the comparisons performed. Statistical analysis available in Genespring software was run. One-way analysis of variance (ANOVA; *p < 0.05*) followed by Tukey’s pairwise comparisons were performed to describe transcriptomic profile differences along the time for each cell type in response to *V. vulnificus* infection.

Transcriptomic data are available at Gene Expression Omnibus (GEO) database with accession number GSE196944.

### Validation of Microarray Results by RT-qPCR

RT-qPCR was performed in parallel to hybridization to validate the microarray results. Table 1 lists the genes, the conditions in which the samples were taken, and the control sample used in each case to calculate the fold induction. Supplementary Table S1 lists the primers used. cDNA samples were obtained from RNA using Maxima H Minus Reverse Transcriptase (Thermo Scientific). Then, qPCR was performed on cDNA using Power SYBR^®^ green PCR Mastermix on a StepOnePlus™ Real-Time PCR System. The CT values were determined with StepOne Software v2.0 to establish the relative mRNA levels of the tested genes, using eel actin (*act*) as the gold standard (Paria et al., 2016) and the fold induction (2^-ΔΔCt^) for each gene was calculated according to Livak and Schmittgen (2001). Statistical analysis was performed using GraphPad Prism 7. Data were analyzed by ANOVA analysis for each gene to determine differences between groups (*p* < 0.05).

**Table 1:** Microarray validation by RT-qPCR.

Gene name	Gene acronym	Sample	FC^1^	
			Array	RT-qPCR
Beta-catenin-like protein 1	*bcl2*	B 3 vs. 0 hpi	1.52 (=)	1.87 (=)
Interleukin 1beta	*il1β*	B 12 vs. 0 hpi	17.16 (++)	23.44 (++)
Interleukin 10 receptor subunit beta	*il10r*	RBCs 3 vs. 0 hpi	4.94 (+)	5.24 (+)
Beta-catenin-like protein 1	*bcl2*	RBCs 12 vs. 0 hpi	−1.90 (=)	−1.03 (=)
p53	*p53*	WBCs 3 vs. 0 hpi	6.76 (+)	7.77 (+)
Interleukin 6 receptor subunit beta precursor	*il6r*	WBCs 12 vs. 0 hpi	6.83 (=)	4.87 (+)

*Comparison of fold change (FC) values obtained by microarray and RT-qPCR. In case of RT-qPCR, results were obtained using act as the reference gene and the fold induction (2^-ΔΔCt^) for each gene was calculated. Primers used are listed in* Supplementary Table S1*. FC value represents the mean obtained from 3 independent biological samples.*

1 *FC: fold change values qualitative classification: =, −2 < X < 2; +, 2 ≤ X < 10; ++, 10 ≤ X < 25; ND, non-detected as differentially expressed.*

### Functional Assays

#### Proteolytic and Hemolytic Activity

Serum samples from infected and control animals were serially diluted in PBS (dilutions from 1:2 to 1:64 were performed). The enzymatic activity of the serum was evaluated by plating 5 μl of the serum samples, and dilutions on 1% agarose plates supplemented with 5% casein (for proteolysis) or with 1% erythrocytes (bovine erythrocytes from Sigma, for hemolysis). 5 μl of PBS and proteinase K (2.5 mg/ml, for proteolysis) or molecular water (for hemolysis) were plated as a negative and positive control for the assay, respectively. Plates were incubated at 28°C for 24 h. The maximal dilution of eel serum with positive activity on agarose-casein or agarose-erythrocytes (transparent halo) was determined and considered the titter of proteolytic and hemolytic activity (Pajuelo et al., 2016). Three independent technical replicates of proteolytic and hemolytic activity were performed for each biological sample of serum.

#### Bacteriolytic Activity

R99 strain was grown in a layout at LB-1 plates. Then, plates were inoculated with 5 μl of the serum samples and dilutions (performed as specified in the previous section). 5 μl of PBS and lysozyme (10^3^ μg/ml) were plated as a negative and positive control for the assay, respectively. Plates were incubated at 28°C for 24 h, and the maximal dilution of eel serum with positive bacteriolytic activity measured as inhibition halo of bacterial growth was determined. Three independent technical replicates of bacteriolytic activity were performed for each biological sample of serum.

### Design and Validation of a New RT-qPCR Assay to the Early Detection of Vv-Vibriosis

A selection of genes (*npsn*, *cox2*, *mmp9* and *sidt1*) have been used to develop a new RT-qPCR assay to the early detection of Vv-vibriosis. The list of primers in Supplementary Table S1. Eel infection, blood sampling, sample processing, and RT-qPCR procedure were performed as described on the previous sections. Statistical analysis was performed using GraphPad Prism 7. Data were analyzed by ANOVA analysis followed by the post-hoc multiple comparison by Bonferroni’s method that was run for each gene to determine differences between groups (*p* < 0.05).

## Results

### Cell Analysis

First, we monitored the presence of the pathogen in blood and found bacterial counts (0 hpi; <10^2^ CFU/ml; 3 hpi; 7 ± 0.5 × 10^2^ ± CFU/ml; and 12 hpi; 3 ± 0.7 × 10^4^ CFU/ml) and cell numbers (RBCs; 1.5 ± 0.3 × 10^9^ at 0 hpi and 2.5 ± 0.5 × 10^9^ cells/ml at 12 hpi: WBCs; 1.7 ± 0.9 × 10^7^ at 0 hpi and 2.3 ± 1.1 × 10^7^ cells/ml at 12 hpi). The bacterial counts and cell numbers found in eel blood in our experiments were similar to those previously obtained from eels infected by immersion (Callol et al., 2015a; Pajuelo et al., 2015) but lower than those obtained from intraperitoneally-infected eels (Valiente et al., 2008b). We highlight the high number of RBCs in the eels from the stock analyzed, values that were similar to those found by Valiente et al. (2008b), compared to those found in the eels’ stock analyzed by Callol et al. (2015a). These apparently contradictory results are not surprising, given that eels do not reproduce in captivity and that researchers work with wild populations of different origins (Palstra et al., 2005; Mes et al., 2016; Jehannet et al., 2017).

### Transcriptomic Analysis

**Figure 1:**
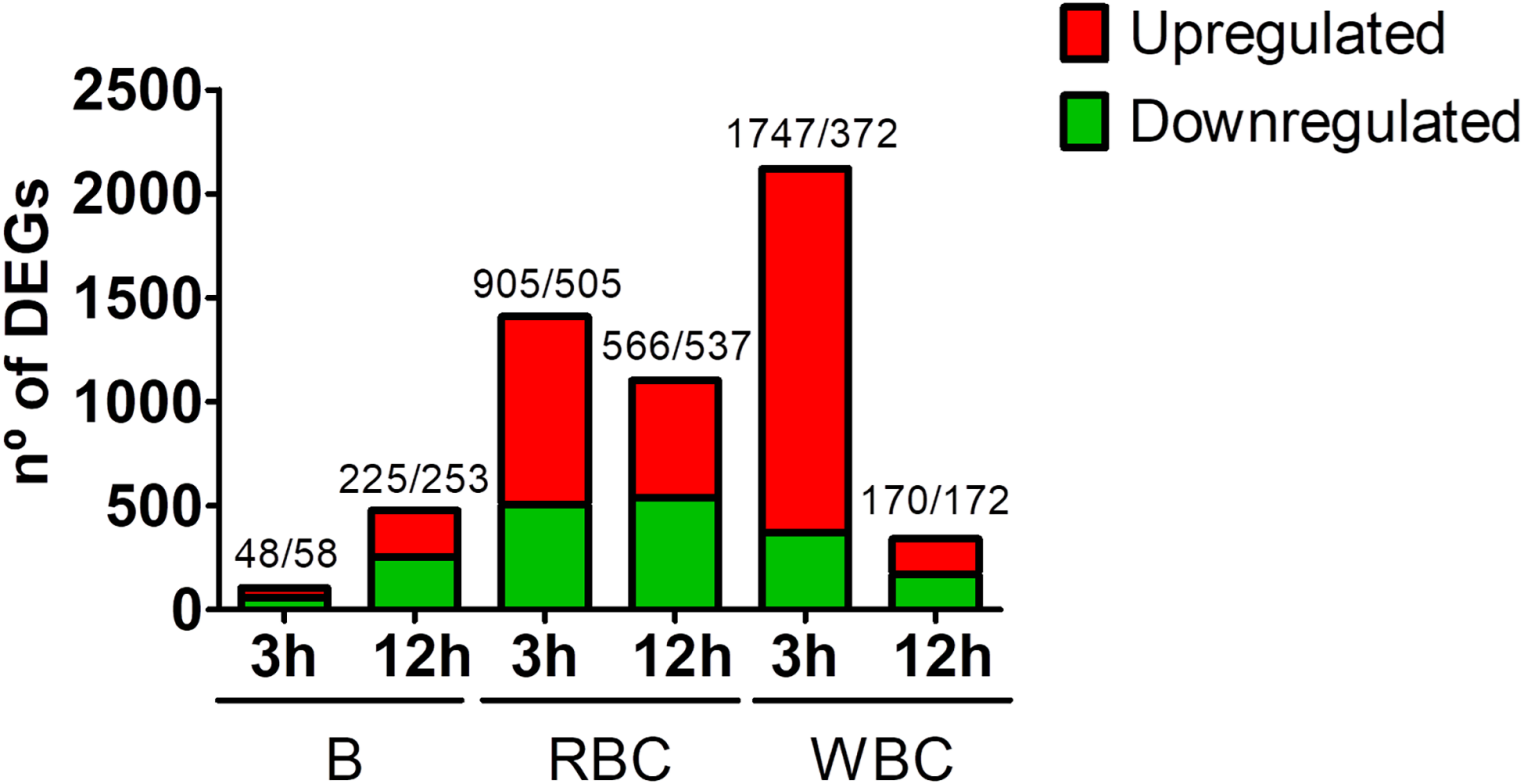
Magnitude of the eel immune response against *V. vulnificus* represented as the number of differentially expressed genes (DEGs) in blood (B), red blood cells (RBCs) and white blood cells (WBCs) samples. Bars represent total DEGs (sum of upregulated [red] and downregulated [green] DEGs) of each sampling point (3 hpi; 12 hpi) against time zero (0 hpi) of each type of sample. The numbers of up/downregulated DEGs are indicated on the top of each bar.

Figure 1 shows the number of differentially expressed genes (DEGs) per sample and sampling time. The early response of WBCs was greater than that of RBCs, both in terms of the number of DEGs and fold change values (Figure 1; Table 2; Supplementary Table S2). Thus, RBCs and WBCs showed about 1,000 and more than 1,700 upregulated genes, respectively. In contrast, the number of upregulated genes decreased significantly at 12 hpi, especially in the case of WBCs (Figure 1). Previous studies with eel RBCs and WBCs showed that erythrocytes, granulocytes, and macrophages could be destroyed by *V. vulnificus in vitro* (Lee et al., 2013; Hernández-Cabanyero et al., 2019), which would be compatible with a reduction in cell number that was not found in the present study. Instead of this reduction, we found that RBCs, and especially WBCs, were less transcriptionally active at 12 hpi, which is compatible with a loss of functionality caused directly or indirectly by the pathogen. Interestingly, the number of DEGs detected in the B samples was much lower than that found in the RBCs and WBCs samples (Figure 1). This apparent anomaly could be explained by the cellular heterogeneity of the blood, which could negatively affect the normalization of the data and be the cause of high outlier removal.

**Table 2:** Eel blood transcriptome after *V. vulnificus* infection.

Gene^1^	FC^2^					
	B		RBCs		WBCs	
	3 hpi	12 hpi	3 hpi	12 hpi	3 hpi	12 hpi
**Pathogen detection and antigen presentation systems**						
**PRR**						
*tlr13*	–	4.3	–	–	–	–
*tlr9a*	–	−2.6	–	–	–	–
*tlr20a*	–	–	4.2	–	9.6	–
*tlr3*	–	–	3.4	–	3.7	–
*tlr21*	–	–	3.1	–	4.9	–
*tlr9b*	–	–	2.2	1.8	–	–
*tlr7*	–	–	–	–	4.1	–
*tlr6*	–	–	–	–	3.0	–
*tlrs5*	–	–	–	–	2.2	–
*tlr20f*	–	–	–	–	−5.5	–
**Antigen presentation**						
*mhcII*	–	2.7	3–1.7	1.5	–	–
*mhcI*	–	–	4.8–2	84.4	5.3–3.2	30.6
AP-1 complex subunit sigma 3	–	–	–	−2.0	3.8	–
AP-1 complex subunit gamma 1	–	–	–	–	2.1	–
**Cathepsins**						
Cathepsin L	–	3.6	–	–	5.6	–
Cathepsin S precursor	–	2.9	1.8	–	–	–
Cathepsin B	–	–	2.5	5.3	–	–
**Pathogen control and destruction**						
**Pathogen growth inhibition**						
Transferrin	–	2.7	2.5–2.1	1.8	–	–
Transferrin receptor (*tfr1*)	–	2.4	–	–	–	–
Aminolevulinic acid	–	2.0	–	–	–	2.1
Hemoglobin subunit	–	–	–	3.7	–	–
Ferritin	–	–	–	2.8–2.1	–	–
Hepcidin	–	–	–	–	10.2	–
**Complement system**						
C5a receptor	–	14.1	–	–	–	–
Complement factor Bf-2	–	6.5–5.3	–	–	–	–
Complement factor B/C2	–	5.9	–	–	2.4	–
Complement factor B	–	4.6	–	–	–	–
C3a receptor 1	–	–	6.5	–	7.5	–
C3c	–	–	4.2	–	4.2	–
C7-1	–	–	4.1	–	3.3	–
C4BPB	–	–	4.1	3.0	7.7	–
C3	–	–	3.9	–	7.4	–
C3-H2	–	–	3.6	–	4.2	–
C3-H1	–	–	3.5	–	2.9	–
C3-S	–	–	3.1	1.9	5.6–2.6	1.9
factor D precursor	–	–	2.8	–	–	–
C4-2	–	–	2.2	–	2.1	2.7
C5	–	–	–	–	12.8	–
C5-2	–	–	–	–	6.1	–
C4	–	–	–	–	4.1	–
C4b	–	–	–	–	4.0	–
C3-3	–	–	–	–	3.6	–
C1R/C1S subunit of Ca2 + −dependent	–	–	–	–	3.4–2.9	–
C7	–	–	–	–	3.0	–
Complement factor I	–	–	–	–	2.3	–
C3 precursor	–	–	–	–	2.2	–
C1q, B chain	–	–	–	–	–	7.7
**Antibacterial effectors**						
Nephrosin (*npsn*)	10.1–6.3	150.6–99	–	–	–	–
Lpb/Bpi	4.5–3.3	41.7–25	–	–	–	–
Nitric oxide synthase	–	–	–	–	3.4	–
**Lectins**						
Mannose-6-phosphate receptor-binding	–	14.0	–	–	–	–
C-type lectin receptor	–	–	3.8	–	11.5-(−6)	–
*gal3*	–	–	3.2	–	4.3	1.7
*gal4*	–	–	2.9	1.7	16.1	–
Intelectin	–	–	2.5	–	12.6	–
Mannose binding lectin 2	–	–	–	3.0	3.1–2.1	–
Fucolectin 2	–	–	–	–	2.7	–
Fucolectin 4	–	–	–	–	2.1	1.9
**Cytoskeleton rearrangements**						
Tubulin-related genes	–	27.2	–	–	2.0	–
Myosin-related genes	–	5.9	7.6–2.6	6–1.6	8.2–2.2	–
*itpr1*, *itpr3*	–	–	4.9–2.7	–	–	–
Actin-related genes	–	3.1	3.4	7–1.5	4.7–2	1.8
Coronin-1a	–	–	2.9	–	–	–
**Cell death**						
**Apoptosis**						
*atg9*	–	–	–	–	3.3	–
*atg2a*	–	–	–	–	3.3	–
Autophagy						
*p53*	–	–	6.8	–	9.9	–
Calpain	–	–	–	−3.9	−4.7	–
p53 apoptosis effector related to PMP-22	–	–	–	–	33.5	–
Apoptosis-inducing factor 3	–	–	–	–	6.8	–
*casp8*	–	–	–	–	3.0	–
*casp3*					–	4.8^*^
**Inflammatory response**						
**Signal transducers and transcriptional factors**						
*klf6*	2.2	8.5–5.2	–	–	–	–
src-family tyrosine kinase SCK	–	17.2	–	–	–	–
*traf3*	–	5.4	–	–	–	–
*socs3*	–	5.1	–	–	–	–
*c-fos*	–	3.7	–	16.9	–	–
*jun-b*	–	3.5	–	11.1	–	–
*map2k6*	–	–	8.6	–	25.3	–
*nod1*	–	–	4.9	–	4.3	–
*p38*	–	–	4.5	4.2	4.0	–
*map3k2*	–	–	4.5	–	4.2	–
*pak1*	–	–	3.3	–	6.9	–
*map3k5*	–	–	3.1	–	6.9	–
*mapk7*	–	–	−2.0	−2.0	–	–
*irak4*	–	–	−2.4	–	–	–
*jak1*	–	–	−2.4	–	–	–
*erk1*	–	–	−1.9	−2.0	–	–
NF-Κβ inhibitor alpha	–	–	–	3.3	−4.8	–
*nod3*	–	–	–	2.8	3.6	–
*klf13*	–	–	–	2.2	–	–
*map3k4*	–	–	–	−2.3	–	–
c-myc binding protein	–	–	–	−5.5	–	–
*mapk6*	–	–	–	–	24.3	–
NF-Κβ p105 subunit	–	–	–	–	4.8	–
Kdel receptor 3	–	–	–	–	4.3	–
*mapk14*	–	–	–	–	4.0	–
*stat3*	–	–	–	–	3.5	3.1
*nlrc3* receptor	–	–	–	–	2.6	–
*cop9*	–	–	–	–	2.1	–
**Inflammatory cytokines and related proteins**						
Interferon induced protein 2	–	20.1	–	–	–	–
*il1β*	–	17.2	–	–	3.5^*^	27.8^*^
Granulin	–	9.3	–	–	–	–
IL8 precursor	–	7.5	–	–	–	–
IL1β receptor type 1 soluble	–	5.5	–	–	−4.2	–
Progranulin type 1	–	5.5	–	–	–	–
IL1 receptor type 1	–	4.4	–	–	−6.2	–
*il12β*	–	–	8.5	–	14.8	–
IL10 receptor β	–	–	4.9	–	22.2	–
*il17a/f1*	–	–	4.4	–	–	–
*il20*	–	–	2.7	–	6.0	–
*irf3*	–	–	2.4	–	–	–
nuclear factor interleukin 3-regulated protein	–	–	–	10.1	3.5	–
IL6 receptor subunit β precursor	–	–	–	6.8	4.3	2.4
*irf2A*	–	–	–	2.7	–	–
*irf2B*	–	–	–	2.2	–	–
IRF2, promoter region	–	–	–	2.2	–	–
Tumor necrosis factor receptor (*tnfrsf12a*)	–	–	–	–	15.9	–
TNF receptor member 27	–	–	–	–	6.0	–
*irf1*	–	–	–	–	3.3–2.8	–
IL1 receptor-like	–	–	–	–	3.1	–
*ifnc1*	–	–	–	–	2.6	–
Allograft inflammatory factor-1	–	–	–	–	2.6	–
TNF receptor associated factor 2	–	–	–	–	2.4	–
*ifna2*	–	–	–	–	2.1	–
*il17r*	–	–	–	–	–	2.2
**Chemokines and receptors**						
CC CK3	–	3.6	–	–	–	–
C-C receptor type 4	–	–	6.2	–	–	–
CK 21 precursor	–	–	4.1	–	–	–
CCL4	–	–	–	–	8.6	13.4
CK 4 precursor	–	–	–	–	8.2	–
CK 19 precursor	–	–	–	–	5.2	–
CK 10 precursor	–	–	–	–	2.2	–
**Septicemia markers**						
Ciclooxigenase-2 (*cox2*)	5.5	32.1	–	–	–	–
Hyaluronidase-2 (*hyal2*)	2.5	26.6	–	–	–	–
*mmp9* or *gelatinase B*	–	61.5–40	–	–	–	–
Leukotriene	–	5.2	–	–	–	–
Prostaglandin	–	11.0	–	–	–	–
**Coagulation factors**						
Coagulation factor VIII	–	22.4–9.1	–	–	–	–
Platelet receptor Gi24	–	2.0	–	–	–	–
Antithrombin protein	–	–	3.8	–	4.1	–
Thrombin protein	–	–	3.4	–	–	–
Thrombospondin	–	–	–	–	7.1–3	–
Coagulation factor V	–	–	–	–	4.6	–
Fibrinogen	–	–	–	–	4.0	–
Angiotensinogen	–	–	–	–	3.3	–
Plasminogen	–	–	–	–	2.0	1.9
Multiple coagulation factor	–	–	–	–	–	3.1
**Angiogenesis and hematopoiesis**						
*angpt2*	–	5.9	–	–	−3.7	–
*angpt1*	–	–	5.6	–	7.4	–
*cldn19*	–	–	3.2	–	11.6	–
*cldn1*	–	–	3.2	–	7.2	–
*cldn18*	–	–	–	–	15.4	–
*cldn4*	–	–	–	–	4.1	–
*cldn29a*	–	–	–	–	3.0	–
**Epigenetic response**						
Histone H2B	–	−2.3	–	–	–	–
Histone H2AFX	–	–	3.2	2.2	–	–
Anti-silencing protein	–	–	2.3	–	3.1	–
Histone acetyltransferase type B catalytic subunit	–	–	2.2	–	2.6	–
Histone deacetylase 3	–	–	2.2	–	–	–
Histone gene cluster XlH3-A (*h1a*, *h2b*, *h3*, *h4*)	–	–	−1.4	–	–	–
Histone acetyltransferase MYST2	–	–	−1.4	–	–	–
Histone H1x	–	–	−3.5	–	–	–
Histone H3.3	–	–	–	1.3	–	–
euchromatic histone-lysine N-methyltransferase 1b (*ehmt1b*)	–	–	–	−1.7	–	–
Histone acetyltransferase MYST4	–	–	–	−2.7	–	–
Histone deacetylase 1	–	–	–	−2.7	–	–
Histone H2A.Z	–	–	–	−4.0	–	–
Histone deacetylase 2	–	–	–	−4.3	–	–
Histone H1	–	–	–	–	7.4–5.9	–
Histone H2AV	–	–	–	–	−1.8	−1.6
**Relationship between systemic and mucosal immunity**						
*muc2A*	–	–	2.5	–	4.4	–
**RNA-based response**						
Systemic RNA deficient-1 (*sidt1*)	–	10.5	–	–	5.4^*^	2.2^*^
**Stress-related response**						
Hypoxia-inducible factor 1 alpha	–	3.5	–	–	–	–
Glutathione peroxidase	–	−2.1	−1.5	−1.7	−2.0	–
*hsp90* (cochaperone activator of Tlr9)	–	–	3.6	5–1.8	36.9–8.2	–
Inositol hexakisphosphate (*insP6*)	–	–	2.0	2.9	–	–
*hsp70*	–	–	–	31.7–2.2	25.2	9.1
Osmotic stress gene	–	–	–	6.7	–	–

*List of selected differentially expressed genes (DEGs) from eels infected with V. vulnificus R99 strain. DEGs are grouped according to their putative biological function. The fold change (FC) values are based on the comparison between the time indicated on top of each column (3 hpi; 12 hpi) compared to time zero (0 hpi) for the same type of sample [B (blood), RBCs (red blood cells), or WBCs (white blood cells)]. FC value represents the mean obtained from 3 independent biological samples*. *–Not detected as differentially expressed*.

1 *Identified DEGs are indicated*.

2 *FC: fold change value for each individual gene. See Supplementary Table S2 for specific gene and fold-change value*.

* *Relevant mRNAs detected by RT-qPCR*.

**Figure 2:**
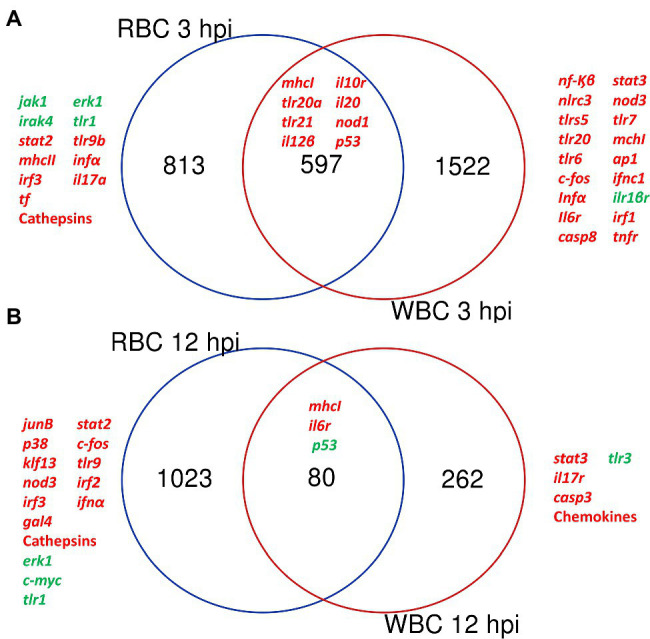
Red blood cells (RBCs) and white blood cells (WBCs) elicit a different immune response against *V. vulnificus*. Venn diagram depicting the overlap of the differentially expressed genes (DEGs) between RBCs and WBCs at 3 hpi **(A)** and 12 hpi **(B)**.

Overall, the transcriptomic results were reliable thanks to the similar fold change values obtained by microarray hybridization and RT-qPCR for a set of genes (Table 1). Venn diagrams showing common DEGs in RBCs and WBCs throughout infection revealed a cell-specific response, as most transcripts that change their transcription level were not shared between both cell types (Figure 2).

The DEGs by the different blood fractions are shown in Supplementary Table S2, and a selection of them by putative function is listed in Table 2. Based on this information, we highlight the following genes and processes that could be related to a harmful defensive response (which could cause self-damage in host tissues and favor its death) of eel against *V. vulnificus*:

#### Pathogen Detection and Antigen Presentation Systems

RBCs and WBCs upregulated multiple pattern recognition receptor (Prr) genes as well as major histocompatibility complex (Mhc) genes, the former mainly at 3 hpi and the latter at both 3 and 12 hpi (Table 2; Supplementary Table S2). This result suggested that not only WBCs but also RBCs could act as antigen-presenting cells. Among the Prr genes, we found upregulated *tlr* genes (Toll-like receptors [Tlrs]), some of which were specifically associated with cell type: i.e., *tlr7*, *tlr6,* and *tlr5s* (encoding the soluble form of Tlr5) with WBCs and *tlr9b* with RBCs (Figure 2; Table 2). Some of these *tlr* genes are related to the detection of mainly extracellular antigens (*tlr20a*, *tlr21*, *tlr6* and *tlr5s*) and others to the detection of intracellular ones (*tlr3*, *tlr7,* and *tlr9b*). Consistent with this, genes encoding major histocompatibility complex (Mhc) class I (*mhcI*) and class II (*mhcII*) were also upregulated, *mhcI* by RBCs and WBCs, and *mhcII* only by RBCs. Thus, although *V*. *vulnificus* is an extracellular pathogen, our results point to activation of intracellular pathogen recognition and processing mechanisms frequently associated with viral infection (Lund et al., 2004).

In all samples (B, RBCs and WBCs), we also found upregulated genes for cathepsins B, L, and S, a group of lysosomal proteases that play a key role in cellular protein turnover. Cathepsins are associated with Tlr signaling pathways in blood cells to the extent that their inhibition blocks Tlr3-, Tlr7,- and Tlr9-mediated responses (Matsumoto et al., 2008).

#### Pathogen Control and Destruction

We found evidence of an antibacterial response in blood of infected eels, suggesting that the host immune response tried to eliminate the pathogen after the infection. Our data showed the upregulation of multiple genes related to pathogen growth inhibition (transferrin [RBCs] and hepcidin [WBCs], the hormone that controls iron sequestration, pathogen tagging (i.e., complement factor C3 and lectins), and pathogen destruction (i.e., complement factors C5-C9, Lbp/Bpi protein and genes related to activation of phagocytosis; Table 2; Supplementary Table S2). Complement genes were upregulated by RBCs and WBCs, especially at 3 hpi, although the strongest and most varied response was associated with WBCs at 3 hpi. Similarly, both cell types’ upregulated genes encoding lectins at 3 hpi, especially the galectin and intelectin. Complement/lectin-tagged bacteria can be recognized and phagocytosed more easily by host phagocytes. In accordance, we found several upregulated genes that could be related to phagocytosis and bacterial killing, such as those involved in cytoskeleton rearrangements and nitric oxide synthesis (i.e.*, inos* that was only upregulated by WBCs at 3 hpi), as well as genes related to signal transduction in common with other cellular processes that will be discussed in the following sections (Table 2; Supplementary Table S2).

Regarding antibacterial activity, our results showed the gene encoding Lbp/Bpi, an antibacterial protein produced by different cell types (Inagawa et al., 2002), to be highly upregulated, but only in B samples. Surprisingly, the gene coding for nephrosin (*npsn*), which has recently been linked to the antibacterial activity of the immune system in fish (Di et al., 2017), was the most strongly upregulated gene in the B samples at both 3 and 12 hpi (Table 2; Supplementary Table S2).

#### Cell Death

We detected upregulation of multiple genes related to the activation of cell death by apoptosis and/or autophagy, but interestingly only in the WBCs fraction with the sole exception of *p53* (RBCs, 3 hpi). Thus, autophagy could be related to the upregulation of autophagy-related proteins (*atg9* and *atg2a*); and apoptosis to *p53*, caspase-8 (*casp8*), and genes encoding apoptosis-inducing factors, all upregulated at 3 hpi (Table 2; Supplementary Table S2).

#### Inflammatory Response

We also found evidence of early regulation of different pathways that trigger a proinflammatory response. At the signal transduction level, this response consisted of upregulation by RBCs and WBCs of nucleotide-binding oligomerization domain containing proteins (*nod1* and *nod3*), as well as the genes for Mapk kinases *map2K6*, *map3K2,* and *map4K5*, whereas *mapK6*, *NF-Κβ* (p105 subunit), and signal transducer and activator of transcription 3 (*stat3*) were only upregulated by WBCs (Table 2; Supplementary Table S2). Indeed, Nod1 and Nod3 activate the Nf-Κβ and Mapk signaling pathways, enhancing the transcription of proinflammatory cytokines (Kim et al., 2016). The rest of the genes mentioned above are part of the Jak/Stat signaling pathway, which is also involved in activating the inflammatory response (Rawlings et al., 2004). Accordingly, we also found upregulated by WBCs at 3 hpi, a gene for an Nlrc3 receptor that acts as a negative regulator of all these processes (Schneider et al., 2013), suggesting an attempt by the immune system to counteract the activation of the inflammatory response against *V. vulnificus*.

The activated proinflammatory response was also evidenced by the early (3 hpi) upregulation of genes for several tumor necrosis alpha (Tnfα) receptors, interleukins (Ils), and their receptors and interferon (Ifn) and related proteins by RBCs and WBCs with a common (*il12β*, *il10 receptor β*, and *il20*) and cell-type-specific pattern (RBCs: *il17a/f1*; WBCs: several Tnf receptors, Il1 receptor-like, *ifnc1*, *ifna2,* and *irf1*) followed by a strong upregulation of genes for Il1β, two receptors for Il1β, Il8 precursor, progranulin and granulin in B samples at 12 hpi (Table 2). Granulins are multifunctional proteins produced after proteolytic processing of progranulin (Bateman et al., 1990) that enhance the production of proinflammatory cytokines such as Tnfα and Il8 (Park et al., 2011). Related to these results, we highlight that Il1β is the main proinflammatory cytokine in both humans and fish (Zou and Secombes, 2016) and that Ifna has been related to the immune response against virus (Zou and Secombes, 2016). Multiple genes for Tnfα- and interferon-induced proteins were also detected in all the samples (Table 2; Supplementary Table S2). In parallel, a few genes encoding for anti-inflammatory cytokine receptors (e.g., *il10r*) were found early upregulated by RBCs and WBCs, which could be interpreted as an attempt by the organism to control the cytokine storm and restore homeostasis.

Since we detected upregulation of the inflammatory gene markers *il1β* and caspase-3 (*casp3*) in B samples and not in WBCs samples, we performed RT-qPCR with the same samples and found both to be upregulated in WBCs (*il1β* at 3 and 12 hpi; *caps3* only at 12 hpi; Table 2).

#### Sepsis Markers

Cells present in B samples upregulated multiple markers of sepsis at 12 hpi. For example, marker genes for disseminated intravascular coagulation, such as those encoding coagulation factor VIII, and genes encoding leukotrienes, prostaglandins, and cyclooxygenase (i.e., *cox2*), all of which are considered markers of the acute phase of the disease (Peters-Golden et al., 2005; Gómez-Abellán and Sepulcre, 2016; Wang et al., 2016). Leukotrienes increase leukocyte accumulation, phagocytic capacity for microbial ingestion and elimination, and the generation of other proinflammatory mediators (Peters-Golden et al., 2005). Cyclooxygenases enhance prostaglandin production (Smith et al., 2000), leading to the induction of the immune response (Gómez-Abellán and Sepulcre, 2016). More importantly, genes for matrix metalloproteinases that are implicated in endothelial damage (i.e., *mmp9*; Pedersen et al., 2015) were among the most overexpressed genes in B samples at 12 hpi (Table 2; Supplementary Table S2). Related to this damage, genes related to endothelial regeneration (angiogenesis) were also upregulated (angiopoietins *angpt2* and *angpt1* in B and RBCs samples, respectively; Table 2; Supplementary Table S2).

#### Epigenetic Response

Several histone-related genes (acetylases, deacetylases, and methyltransferases, among others) were found to be DEGs (both up- and downregulated) mainly by RBCs (Table 2; Supplementary Table S2). This effect could be associated with an epigenetic response probably related to modulation of the immune response by gene silencing through methylation (Medzhitov and Horng, 2009; Shakespear et al., 2011). In parallel, we also detected a strongly upregulated gene for an anti-silencing protein in RBCs and WBCs samples at 3 hpi (Table 3; Supplementary Table S2). Anti-silencing proteins are evolutionarily conserved proteins that act as histone chaperones and are required for various chromatin-mediated cellular processes. Recently, it has been demonstrated that all these proteins are involved in antiviral mechanisms promoting Ifnb production (Liu et al., 2016).

#### Relationship Between Systemic and Mucosal Immunity

It was not surprising to find DEGs that evidenced a link between systemic and mucosal immunity that our group had previously shown to occur in eels vaccinated against *V. vulnificus* (Esteve-Gassent et al., 2003). Among them, it should be highlighted *muc2A*, a gene for a mucolipin secreted by mucosal cells that is involved in binding to bacteria for killing (McGuckin et al., 2011; Brinchmann, 2016) and that was upregulated at 3 hpi by both RBCs and WBCs (Table 2; Supplementary Table S2).

#### RNA-Based Response

One of the most striking results of the present study was the strong upregulation by B samples of a gene for a specific transporter of a systemic interference RNA, *sidt1* (systemic RNAi deficient-1; Li et al., 2015), which was detected at 12 hpi (Table 2). This result strongly suggested that a systemic RNAi may be acting during the immune response against *V. vulnificus.* It is well known that systemic RNAi, common to all vertebrates, is involved in ancestral innate defense mechanisms against viral infections (Li et al., 2015). Since we detected upregulation of this gene in the B samples and not in WBCs samples, we performed RT-qPCR on the same samples and found the gene to be upregulated in WBCs at 3 and 12 hpi (Table 2).

### Functional Assays

The transcriptomic results were confirmed by evaluating different enzymatic and lytic activities in eel serum samples. We detected proteolytic, hemolytic, and bacteriolytic activities in serum that were significantly increased at 3 hpi and 12 hpi compared to those found in serum samples at 0 hpi and those found in serum samples from non infected animals (Table 3).

**Table 3:** Proteolytic, hemolytic and bacteriolytic activity of eel serum before and after *V. vulnificus* infection.

Serum sample	Proteolytic activity^1^	Hemolytic activity^2^	Bacteriolytic activity^3^
Non-infected	–	1:2	1:2
0 hpi	–	1:8	1:4
3 hpi	1:8	1:8	1:4
12 hpi	1:4	1:4	1:8

*Eels were infected by immersion and the lytic activities were determined in serum from non-infected eels (control), and eels infected at different hours post infection (hpi). Results are presented as the titter (maximal dilution of serum with a positive result in 3 independent biological samples) of the corresponding activity*. –Without activity.

1 Proteolytic activity: evaluated by plating 5 μl of the serum samples and dilutions (serial dilution 1:2 to 1:64 on PBS) on 1% agarose plates supplemented with 5% casein. The maximal serum dilution that produced a transparent halo was considered as the titter of this activity.

2 Hemolytic activity: evaluated by plating 5 μl of the serum samples and dilutions (serial dilution 1:2 to 1:64 on PBS) on 1% agarose plates supplemented with 1% erythrocytes (bovine erythrocytes from Sigma). The maximal serum dilution that produced a transparent halo was considered as the titter of this activity.

3 Bacteriolytic activity: evaluated by plating 5 μl of the serum samples and dilutions (serial dilution 1:2 to 1:64 on PBS) on LB-1 plates inoculated with a *V. vulnificus* lawn. The maximal serum dilution that inhibited bacterial growth was considered as the titter of this activity.

### Early Diagnosis of Fish Septicemia by RT-qPCR

The use of selected gene markers for the early detection of fish septicemia was evaluated by RT-qPCR from eels infected by immersion with *V. vulnificus*. To do so, we selected the most upregulated genes related with antibacterial activity (*npsn*), endothelial damage and acute phase of infection (*cox2* and *mmp9*) and the transporter of a systemic interference RNA (*sidt1*; Table 2). We infected eels with *V. vulnificus* and analyzed the expression of the selected genes in blood of the infected animals at 3 and 12 hpi compared to non-infected eels. All the selected genes were easily detected upregulated in blood of the infected animals, especially *cox2* and *sidt1* at 3 hpi (Table 4). Therefore, we propose that this easy and fast methodology could be used to the early diagnose of Vv-vibriosis.

**Table 4:** Early diagnosis of fish vibriosis due to *V. vulnificus* by RT-qPCR.

Gene name	Gene acronym	3 hpi	12 hpi
Nephrosin	*npsn*	4.2 (+)	1.5 (=)
Cyclooxygenase 2	*cox2*	21.9 (++)	10.5 (++)
Matrix metalloproteinase-9	*mmp9*	3.1 (+)	2.9 (+)
Systemic RNAi deficient-1	*sidt1*	11.31 (++)	2.5 (+)

Blood samples taken at 3 and 12 hpi from infected and control animals were used to determine the expression of genes selected as septicemic markers by RT-qPCR. Results were obtained using *act* as the reference gene and the fold induction (2^-ΔΔCt^) for each gene was calculated. Primers used are listed in Supplementary Table S1. Fold induction values qualitative classification: =, −2 < X < 2; +, 2 ≤ X < 10; ++, 10 ≤ X < 25.

## Discussion

*V. vulnificus* is an emerging zoonotic pathogen associated with fish farms as all clonal groups defined in the species have emerged from outbreaks of fish vibriosis in farms and contain clinical isolates from fish and humans (Roig et al., 2018; Carmona-Salido et al., 2021). Interestingly, this species uses both generalist and host-specific virulence mechanisms, the former mainly related to its toxins and exoenzymes, and the latter to resistance to innate immunity (Hernández-Cabanyero et al., 2019). Using both, *V. vulnificus* can survive and cause rapid death by septicemia in hosts as evolutionarily distant as humans and eels. Previous studies using mice as an animal model suggested that sepsis death of their original hosts may be due to an early cytokine storm triggered by the pathogen during its interaction with the immune system (Murciano et al., 2017). In this work, we set out to demonstrate this hypothesis using one of the natural hosts of the disease, the eel, and reproducing the natural conditions of infection with a representative strain of the most studied zoonotic group. For the study, a microarray platform was used that was designed from the transcriptome of the hematopoietic organs of eels stimulated with viral/bacterial PAMPs and was consequently enriched in immune genes (Callol et al., 2015b).

First, we highlight the critical role that eel RBCs appear to play in the defense against *V. vulnificus* and, probably, against bacterial pathogens in general. We suspected that RBCs were immunologically active cells because we had observed that bacteria agglutinated in the presence of eel erythrocytes *in vitro* (Lee et al., 2013). In this work, we found that RBCs do indeed activate multiple lectin genes in response to *V. vulnificus* infection that could exert this antibacterial function. We also found that RBCs are genetically primed to act as antigen-presenting cells, as they also activate the transcription of extracellular and intracellular Prrs (Tlrs and Nods), as well as Mhc classes I and II. Similar results were previously found in rainbow trout RBCs which express *mhcII* in response to virus (Nombela et al., 2019). In addition, they are genetically prepared to produce proinflammatory cytokines such as Il17 and Il20 as well as Il12β, whose hypothetic function will be commented on later (Rutz et al., 2014; Zou and Secombes, 2016). Although demonstrating that RBCs act as antigen-presenting cells or produce these cytokines are beyond the scope of this work, these results are consistent with what we know about eel vibriosis, and with the results we have obtained when analyzing the other blood fractions.

Thus, eel WBCs also appear to be very active during the first hours of infection, upregulating the transcription of Prr genes for extracellular and intracellular antigens and, interestingly, only MhcI, the form of Mhc associated with intracellular antigen presentation. In this regard, our results suggest that both RBCs and WBCs may overexpress Tlrs that in fish detect double-stranded RNA (Tlr3 and Tlr13) and DNA (Tlr9 and Tlr21) both extracellularly (Tlr21) and intracellularly (Tlr3, Tlr9, and Tlr13), again suggesting that *V. vulnificus* could be recognized and processed as if it was an intracellular pathogen. We also observed that eel WBCs could produce an orthologue of mammalian Tlr6 whose function is unknown, as it has not been previously described in any fish species. As expected, we also found considerable evidence that eel WBCs could produce numerous antibacterial compounds and act as phagocytic cells, especially in the short term after infection.

Our transcriptomic results also suggest that signaling pathways would converge in RBCs and WBCs on Iraq4 and Traf3, consistent with activation at 3 hpi of an atypical proinflammatory response typically antiviral (Table 2; Supplementary Table S2). Thus, RBCs and WBCs activated the transcription of genes for Il12β, Il17, and Il20 and several genes for type 1 interferons. These interleukins have been linked to mucosal inflammation in mice and humans, especially in inflammatory bowel diseases (Rutz et al., 2014; Zou and Secombes, 2016; Moschen et al., 2019), while Il12β has been linked to antiviral response in both fish and humans (Sakai et al., 2021). This result is very interesting. Firstly, because links systemic and mucosal immunity, which correlates with previous results showing that eels vaccinated *via* mucosal route, produce both mucosal and systemic antibodies against *V. vulnificus* that protect them against Vv-vibriosis (Fouz et al., 2001; Esteve-Gassent et al., 2003). Secondly, the production of Il12β, interferon type 1, and their regulators together with the activation of genes for intracellular antigen recognition and processing mentioned above strongly evidence that this pathogen could be recognized as if it was an intracellular pathogen. Finally, we also found strong evidence that this atypical early immune response leads to a typical inflammatory response at 12 hpi, with upregulation of *il1β*, *il8,* and the *il1βr* that were detected in B and WBCs samples.

In parallel to all these processes, cell death mechanisms by autophagy and apoptosis are probably activated, especially in WBCs. At the same time, RBCs mainly would suffer a stressful situation, as indicated by the strong upregulation of stress markers (Table 2; Supplementary Table S2). Although an increase in the number of WBCs occurs as a natural response in bacterial infections, we did not observe this proliferation in response to *V. vulnificus,* which would be compatible with death by apoptosis or autophagy of a fraction of WBCs. Related to this, we also observed a drastic reduction in the transcription of most of the genes that had been upregulated at 3 hpi. In contrast, RBCs changed their transcriptional pattern by stopping to transcribe genes for proinflammatory cytokines and chemokines and transcribing genes for MhcI, c-Fos, JunB, Irf2A, Irf2B, and cathepsin B. An overproduction of c-Fos, JunB, and cathepsin B has been linked in fish to tissue repair and the over-activation of *irf2A* and *irf2B* with inhibition of interferons alpha and beta (Sato et al., 2009; Botwright et al., 2021), both processes probably related with an attempt to control the strong immune response that was activated at 3 hpi.

Thus, the pathogen would activate an atypical cytokine storm at 3 hpi, followed by a strong inflammation at 12 hpi and blood cell stress and death. This strong inflammation could also lead to endothelial destruction, evidenced by a significant strong activation of sepsis markers related to this destruction; a result compatible with natural disease given that this disease is known as hemorrhagic septicemia (Ince et al., 2016). Beneath this inflammatory response, we found evidence for the activation of a systemic RNAi. Systemic RNAi are part of the conserved biological response mechanisms to double-stranded RNA and are involved in resistance to endogenous and exogenous pathogenic nucleic acids (Abubaker et al., 2014). Its function in fish innate immunity is entirely unknown. Taking all the above mentioned results into account, we hypothesized that *V. vulnificus* could activate a response against endogenous RNA that, in turn, would trigger the cytokine storm. In fact, it has been recently published the activation of this kind of response in patients with sepsis (Chousterman et al., 2017). Further, we hypothesized that the toxin RtxA1 would be one of the responsible virulence factors.

Previous studies demonstrated that mutants deficient in this toxin kept the ability to infect and invade the bloodstream but were unable to cause death by sepsis in fish while were attenuated in virulence and unable to activate the early cytokine storm in mice (Lee et al., 2013; Murciano et al., 2017). Similarly, in human immune cells, the RtxA1 toxin enhances inflammatory pathways (Kim et al., 2020). In addition, this toxin has an intracellular existence as it is secreted after contact with the eukaryotic cell, associates with the cell membrane for its terminal ends, and forms a pore that allows the central module to enter, self-process, and release the functional domains that attack the cell (Satchell, 2015). Studies are in progress to demonstrate this hypothesis.

**Figure 3:**
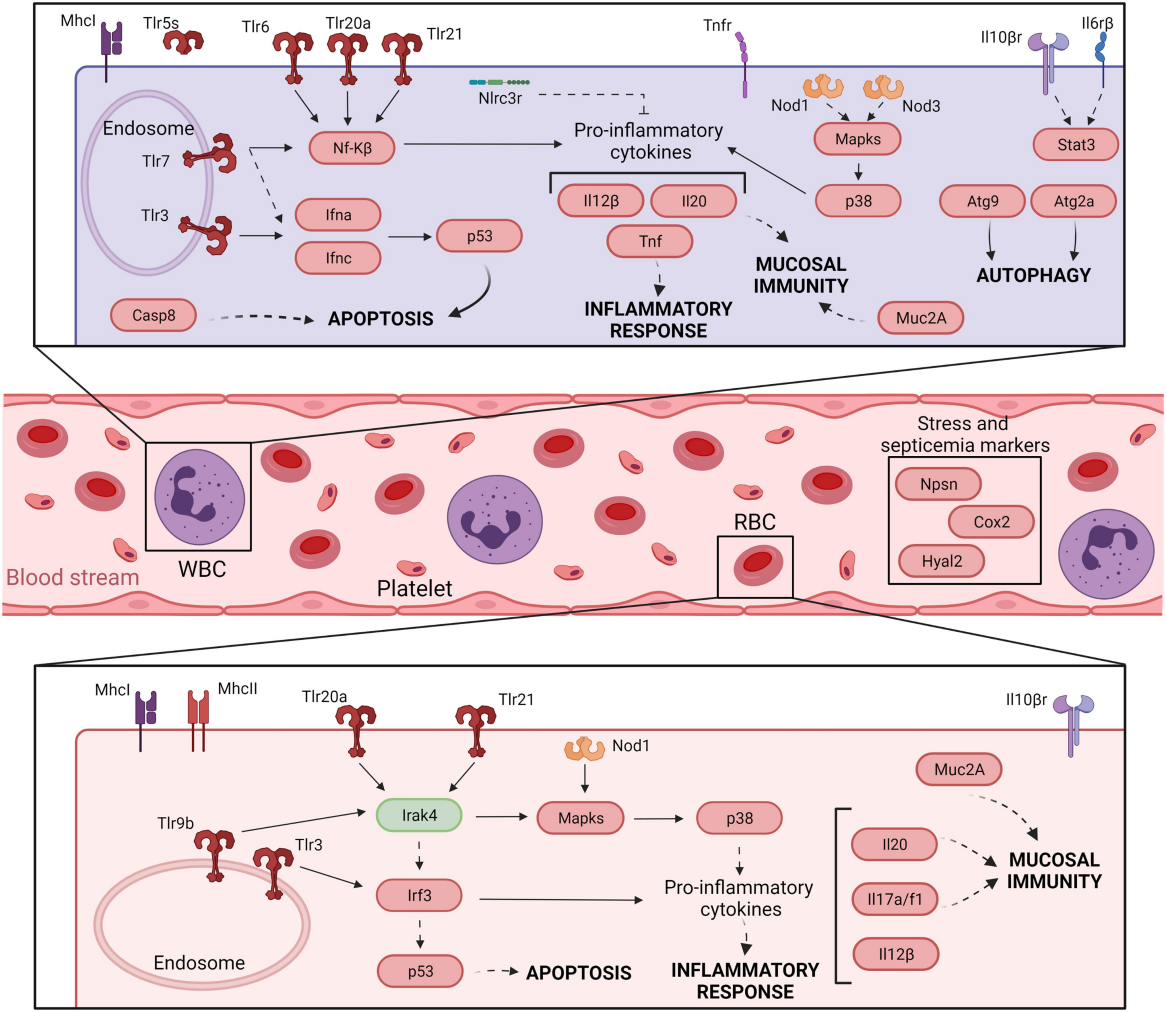
Model of the immune response in eel blood against *V. vulnificus* infection: early phase of vibriosis (3 hpi). The model shows the resultant proteins produced by the main transcripts differentially expressed by eels’ blood cells during the early phase of Vv-vibriosis (at 3 hpi with *V. vulnificus* R99 strain) infection. The putative translated proteins from the major immune-related pathways are represented in a code color depending on the gene modulation: upregulated (red) and downregulated (green).

**Figure 4:**
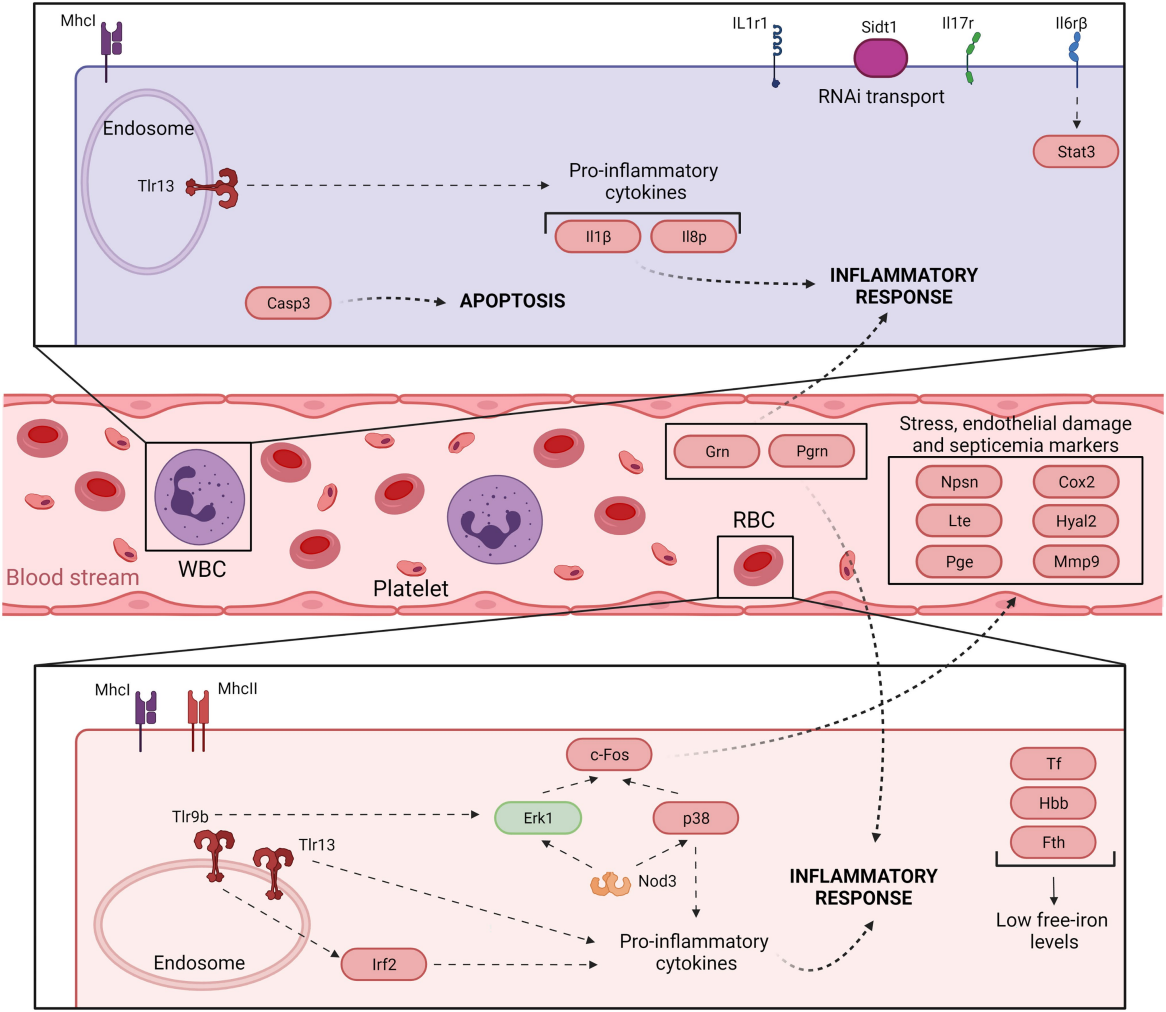
Model of the immune response in eel blood against *V. vulnificus* infection: late phase of vibriosis (12 hpi). The model shows the resultant proteins produced by the main transcripts differentially expressed by eels’ blood cells during the late phase of Vv-vibriosis (at 12 hpi with *V. vulnificus* R99 strain) infection. The putative translated proteins from the major immune-related pathways are represented in a code color depending on the gene modulation: upregulated (red) and downregulated (green).

Our concluding remarks are summarized in Figures 3 **and** 4, which present a model of the immune response against *V. vulnificus* that sheds light on the comprehension of the disease caused by this zoonotic pathogen in its hosts. It should be noticed that although mammalian RBCs are not nucleated and thus considered not active during the immune response, a recent study has demonstrated that human and murine RBCs are involved in the innate immune response to virus (Lam et al., 2021). Therefore, the proposed model could potentially be extended to all *V. vulnificus* hosts, including humans. According to our model, *V. vulnificus* indeed triggers an acute but atypical inflammatory response that occurs in two main phases. In the early phase (3 hpi; Figure 3), the pathogen triggers the upregulation of a series of proinflammatory cytokine genes related to the mucosal immune response (*il17a*/*f1* and *il20*) along with antiviral cytokine genes (*il12β*) and antiviral factors (*ifna* and *ifnc*), while in the late phase (12 hpi; Figure 4) the upregulation of genes for typical inflammatory cytokines (*il1β*), endothelial destruction (*mmp9* and *hyal2*) and, interestingly, genes related to an RNA-based immune response. Remarkably, some of these genes, especially the gene for systemic RNAi transporter (*sidt1*), could be used for the early detection of septicemia caused by *V. vulnificus* infection, as we could diagnose it from blood samples from artificially infected eels by using an RT-qPCR targeting this gene. However, this proposal should be validated with other fish species and by reproducing the vibriosis caused by other *Vibrios* to determine whether this gene marker is exclusive of Vv-vibriosis. Functional assays also highlighted that the serum from infected animals is proteolytic, hemolytic, and bacteriolytic, partially confirming the transcriptomic results. Finally, we found considerable evidence that RBCs are transcriptionally active and that they may contribute significantly to this atypical immune response, especially in the short term.

## Data Availability Statement

The datasets presented in this study can be found in online repositories. The names of the repository/repositories and accession number(s) can be found at: Gene Expression Omnibus - GSE196944.

## Ethics Statement

All assays involving animals were approved by the Institutional Animal Care and Use Committee and the local authority (Conselleria de Agricultura, Medio Ambiente, Cambio Climático y Desarrollo Rural. Generalitat Valenciana) to use eel for scientific research purposes under the protocol 2016-USC-PEA-00033 type 2. The experiments were carried out following the European Directive 2010/63/EU and the Spanish law “Real Decreto” 53/2013.

## Author Contributions

CA conceived the study and performed the initial design that CH-C improved. CH-C, ES, and FER-L performed the laboratory experiments. CH-C and EV-V analyzed the data. CH-C wrote the first draft of the manuscript that was corrected and improved by CA. CA and CH-C built the final version taking into account all the corrections and suggestions of the other authors. All authors read and approved the submitted version.

## Funding

This work has been financed by grants AGL2017-87723-P co-funded with FEDER funds) from the Ministry of Science, Innovation, and Universities (Spain) and AICO/2018/123 and AICO/2020/076 from Generalitat Valenciana (Spain). CH-C has been financed by grant BES-2015-073117, an FPI grant from the Ministry of Science, Innovation and Universities (Spain). EV-V and FER-L thank the support of Fondecyt iniciación (project number 11221308) and Fondecyt regular (project number: 1211841) (Agencia Nacional de Investigación y Desarrollo (ANID), Government of Chile) grants, respectively. This work was also supported by Ministerio de Ciencia e Innovación (MICIN/AEI) (DOI ID: 10.13039/501100011033), PID2020-120619RB-I00 to CA.

## Conflict of Interest

The authors declare that the research was conducted in the absence of any commercial or financial relationships that could be construed as a potential conflict of interest.

## Publisher’s Note

All claims expressed in this article are solely those of the authors and do not necessarily represent those of their affiliated organizations, or those of the publisher, the editors and the reviewers. Any product that may be evaluated in this article, or claim that may be made by its manufacturer, is not guaranteed or endorsed by the publisher.

## Supplementary Material

Supplementary Figure S1 Experimental design used in this study and comparisons performed in the transcriptomic analysis. For specific information about procedures see Materials and methods section.
