# Corrigendum: A Transcriptomic Study Reveals That Fish Vibriosis Due to the Zoonotic Pathogen *Vibrio vulnificus* Is an Acute Inflammatory Disease in Which Erythrocytes May Play an Important Role

**DOI:** 10.3389/fmicb.2022.942624

**Published:** 2022-05-30

**Authors:** Carla Hernández-Cabanyero, Eva Sanjuán, Felipe E. Reyes-López, Eva Vallejos-Vidal, Lluis Tort, Carmen Amaro

**Affiliations:** ^1^Instituto Universitario de Biotecnología y Biomedicina (BIOTECMED), Universitat de València, Valencia, Spain; ^2^Centro de Biotecnología Acuícola, Facultad de Química y Biología, Universidad de Santiago de Chile, Santiago, Chile; ^3^Department of Cell Biology, Physiology, and Immunology, Universitat Autònoma de Barcelona, Bellaterra, Spain; ^4^Facultad de Medicina Veterinaria y Agronomía, Universidad de Las Américas, Santiago, Chile

**Keywords:** *Vibrio vulnificus*, zoonotic pathogen, blood, erythrocytes, European eel, host-pathogen relationship, immune response

In the original article, we neglected to include the funder “Ministerio de Ciencia e Innovación (MICIN/AEI) (DOI ID: 10.13039/501100011033), PID2020-120619RB-I00 to Carmen Amaro.” The corrected Funding statement appears below:

## Funding

This work has been financed by grants AGL2017-87723-P co-funded with FEDER funds) from the Ministry of Science, Innovation, and Universities (Spain) and AICO/2018/123 and AICO/2020/076 from Generalitat Valenciana (Spain). CH-C has been financed by grant BES-2015-073117, an FPI grant from the Ministry of Science, Innovation and Universities (Spain). EV-V and FER-L thank the support of Fondecyt iniciación (project number 11221308) and Fondecyt regular (project number: 1211841) (Agencia Nacional de Investigación y Desarrollo (ANID), Government of Chile) grants, respectively. This work was also supported by Ministerio de Ciencia e Innovación (MICIN/AEI) (DOI ID: 10.13039/501100011033), PID2020-120619RB-I00 to CA.

The authors apologize for this error and state that this does not change the scientific conclusions of the article in any way. The original article has been updated.

## Publisher's Note

All claims expressed in this article are solely those of the authors and do not necessarily represent those of their affiliated organizations, or those of the publisher, the editors and the reviewers. Any product that may be evaluated in this article, or claim that may be made by its manufacturer, is not guaranteed or endorsed by the publisher.

