# Supplementary material for: A Transcriptomic Study Reveals That Fish Vibriosis Due to the Zoonotic Pathogen Vibrio vulnificus Is an Acute Inflammatory Disease in Which Erythrocytes May Play an Important Role
Source: Front Microbiol. 2022 Apr 1;13:852677. doi: 10.3389/fmicb.2022.852677 (PMC9011161; doi:10.3389/fmicb.2022.852677)
Supplement: Supplementary file 1 [file Table_1.DOCX]

**Table S1. Primers used for RT-qPCR analysis.**

| **Gene name** | **Accession number** | **Gene acronym** | **Sequence** |
| --- | --- | --- | --- |
| Actin | GBXM01001652.1 | *act* | Fw: GACATGGAGAAGATCTGGCA  Rv: GTCAGGATCTTCATGAGGTAGTC |
| Beta-catenin-like protein 1 | GBXM01021159.1 | *bcl2* | Fw: CGCGAGATGACGTCCCAGGT  Rv: GGCTTGGAAGGGCTGTGTGC |
| Interleukin 1beta | GBXM01022553.1 | *il1β* | Fw: CGTGCCACGTGCTCTCACAA  Rv: CAGCACCACCTAGTGGCTGAACC |
| Systemic RNAi deficient-1 | GBXM01014494.1 | *sidt1* | Fw: CTCGCCAACTTGGAACATTT  Rv: GTGACAGGGAGAGGGTGTGT |
| Interleukin 10 receptor subunit beta | GBXM01070147.1 | *il10r* | Fw: GAGCACCTACGGCTGTCACG  Rv: TCCAGCTCTCTCTAGGCTTTG |
| Anti-silencing protein 1 | GBXM01103559.1 | *asf1* | Fw: ACGAGTACACGGACCCAGAG  Rv: TTCTCGCAGTCCTCCATCTT |
| p53 | GBXM01014247.1 | *p53* | Fw: GGAAGGCTGCACGGATCTC  Rv: TCTGCGAACCAGCGATTAAA |
| Interleukin 6 receptor subunit beta precursor | GBXM01019769.1 | *il6r* | Fw: CTCTCTCATCCCGTCCTCAG  Rv: ATAGCACTGCTGCTCCTGGT |
| Nephrosin | GBXM01003546.1 | *npsn* | Fw: CAAGGTGGAGGACAGGTTGT  Rv: AGGGGGTTCCCAGATTATTG |
| Cyclooxygenase 2 | GBXM01009885.1 | *cox2* | Fw: GTACAGCTCCACTGCGTCAA  Rv: CCAACTCCATTGAACACACG |
| Matrix metalloproteinase-9 | GBXM01012580.1 | *mmp9* | Fw: GATCCACAGTCCTCGCTCTC  Rv: GATCCACAGTCCTCGCTCTC |
| Caspase 3 | GBXM01019295.1 | *casp3* | Fw: CCCAGCCCCTTGAATGTTT |
|  |  |  | Rv: CGCGATGGCACAGACGTA |
