# Supplementary figures and images for: A Transcriptomic Study Reveals That Fish Vibriosis Due to the Zoonotic Pathogen Vibrio vulnificus Is an Acute Inflammatory Disease in Which Erythrocytes May Play an Important Role
Source: Front Microbiol. 2022 Apr 1;13:852677. doi: 10.3389/fmicb.2022.852677 (PMC9011161; doi:10.3389/fmicb.2022.852677)

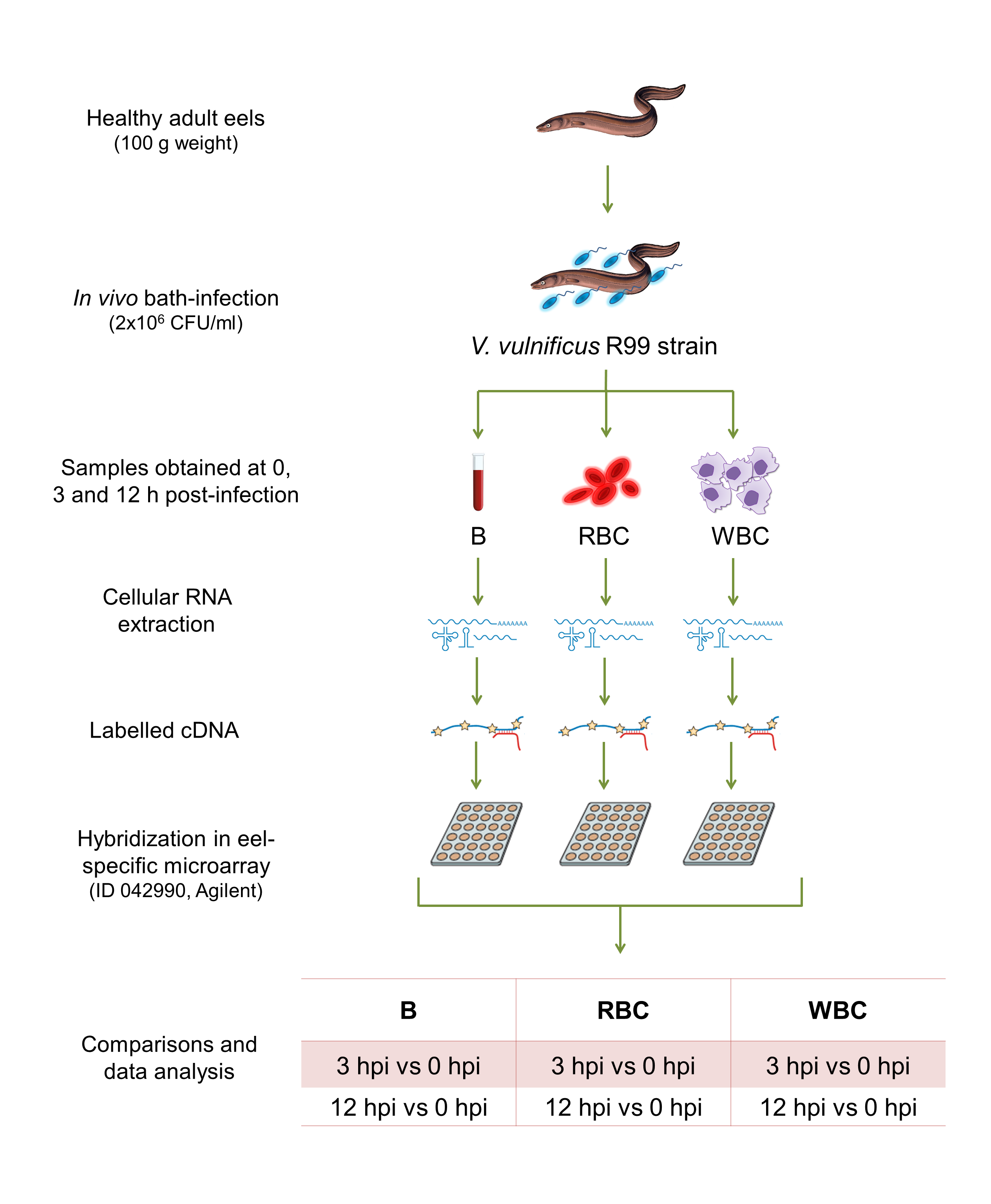

Supplement: Supplementary Figure S1 — Experimental design used in this study and comparisons performed in the transcriptomic analysis. For specific information about procedures see Materials and methods section. [file Image_1.TIF]
